# Do HIV treatment eligibility expansions crowd out the sickest? Evidence from rural South Africa

**DOI:** 10.1111/tmi.13122

**Published:** 2018-07-26

**Authors:** Sheryl A. Kluberg, Matthew P. Fox, Michael LaValley, Deenan Pillay, Till Bärnighausen, Jacob Bor

**Affiliations:** ^1^ Department of Epidemiology Boston University School of Public Health Boston MA USA; ^2^ Department of Global Health Boston University School of Public Health Boston MA USA; ^3^ Health Economics and Epidemiology Research Office Department of Internal Medicine School of Clinical Medicine Faculty of Health Sciences University of the Witwatersrand Johannesburg South Africa; ^4^ Department of Biostatistics Boston University School of Public Health Boston MA USA; ^5^ Africa Health Research Institute Durban and Somkhele KwaZulu‐Natal South Africa; ^6^ Division of Infection and Immunity University College London London UK; ^7^ Institute of Public Health University of Heidelberg Heidelberg Germany

**Keywords:** HIV/AIDS, antiretroviral therapy, guidelines, South Africa, continuity of care, adults, thérapie antirétrovirale, directives, Afrique du Sud, continuité des soins, adultes

## Abstract

**Objective:**

The 2015 WHO recommendation to initiate all HIV patients on antiretroviral therapy (ART) at diagnosis could potentially overextend health systems and crowd out sicker patients, mitigating the policy's impact. We evaluate whether South Africa's prior eligibility expansion from CD4 ≤ 200 to CD4 ≤ 350 cells/μl reduced ART uptake in the sickest patients.

**Methods:**

Using data on all patients presenting to the Hlabisa HIV Treatment and Care Programme in KwaZulu‐Natal from April 2010 to June 2012 (*n* = 13 809), we assessed the impact of the August 2011 eligibility expansion on the number of patients seeking care, number initiating ART and time from HIV diagnosis to ART initiation among patients always eligible (CD4 0–200), newly eligible (CD4 201–350) and not yet eligible by CD4 count (>350). We used interrupted time series methods to control for long‐run trends and isolate the effect of the policy.

**Results:**

Expanding ART eligibility led to an increased number of patients initiating ART per month [+95.5; 95% CI (−1.3; 192.3)]. Newly eligible patients (CD4 201–350) initiated treatment 47% faster than before (95% CI 19%; 82%), while the sickest patients (CD4 ≤ 200) saw no decline in the monthly number of patients initiating treatment or the rate of treatment uptake.

**Conclusion:**

The Hlabisa programme successfully extended ART to patients with CD4 ≤ 350 cells/μl, while ensuring that the sickest patients did not experience delays in ART initiation. Treatment programmes must be vigilant to maintain quality of care for the sickest as countries move to treat all patients irrespective of CD4 count.

## Abstract (fr)

**Objectif:**

La recommandation 2015 de l'OMS d'initier tous les patients VIH à la thérapie antirétrovirale (ART) dès le diagnostic pourrait potentiellement surpasser les systèmes de santé et évincer les patients plus malades, atténuant ainsi l'impact de la politique. Nous avons voulu évaluer si l'expansion préalable de l’éligibilité en l'Afrique du Sud, passant de CD4 ≤ 200 à CD4 ≤350 cellules/μL, réduisait l'adoption de l'ART chez les patients les plus malades.

**Méthodes:**

En utilisant des données sur tous les patients du programme Hlabisa de traitement et de soins du VIH dans le KwaZulu‐Natal d'avril 2010 à juin 2012 (n = 13 809), nous avons évalué l'impact de l'expansion de l’éligibilité en août 2011 sur le nombre de patients recherchant des soins, le nombre débutant l'ART et le temps écoulé entre le diagnostic du VIH et le début de l'ART parmi les patients toujours éligibles (CD4<200), nouvellement éligibles (201<CD4<350) et non encore éligibles (CD4>350). Nous avons utilisé des méthodes de séries temporelles interrompues pour contrôler pour les tendances à long terme et isoler l'effet de la politique.

**Résultats:**

L'expansion de l’éligibilité a entraîné une augmentation du nombre de patients débutant l'ART [+95.5; IC95% (−1.3; 192.3)]. Les patients nouvellement éligibles (201 < CD4 < 350) ont débuté le traitement 47% plus vite qu'avant (IC95%: 19%; 82%), tandis que les patients les plus malades (CD4 ≤200) n'ont pas vu diminuer le nombre mensuel débutant le traitement ou le taux de l'adoption du traitement.

**Conclusion:**

Le programme Hlabisa a étendu l'ART avec succès chez les patients avec CD4 ≤350 cellules/μL tout en veillant à ce que les patients les plus malades ne subissent pas de retards dans l'initiation de l'ART. Les programmes de traitement doivent être vigilants afin de maintenir la qualité des soins pour les personnes les plus malades à mesure que les pays commencent à traiter tous les patients sans tenir compte du nombre de CD4.

## Introduction

In September 2015, the WHO revised its antiretroviral therapy (ART) treatment guidelines, calling for a ‘test‐and‐treat’ strategy, extending treatment eligibility to all people diagnosed with HIV regardless of CD4 count. This recommendation reversed earlier guidelines that limited treatment to patients with lower CD4 counts or severe illness 1. Although expanding eligibility is expected to reduce morbidity, mortality and transmission among patients with high CD4 2, 3, 4, 5, 6, 7, 8, it is possible that a large influx of newly eligible patients in a resource‐limited health system could crowd out sicker patients and reduce quality of care for all patients. As of September 2016, South Africa has joined three other countries in sub‐Saharan Africa in adopting the WHO ‘test‐and‐treat’ policy 9, 10, and additional resource‐limited countries are also considering expanding eligibility 11, 12. To assess the potential for negative spillover effects, we evaluated whether the largest previous eligibility expansion in South Africa affected entry into care and ART uptake among the sickest patients, whose eligibility was not affected but who were exposed to clinic congestion as a result of the guideline change.

South Africa has the largest HIV‐infected population in the world, with 2015 estimates of 7 million people living with HIV and over 3.3 million receiving ART 13. In August 2011, South Africa's National Department of Health extended ART eligibility to all adults with CD4 counts ≤350 cells/μl 14, as recommended by the WHO 2010 guidelines 15. Prior guidelines limited eligibility to patients with CD4 counts ≤200 cells/μl and to pregnant or tuberculosis‐infected patients with counts ≤350 cells/μl. The 2011 policy change was estimated to have increased the ART‐eligible population in South Africa by 900 000 individuals or 51% 16. If the resulting clinic congestion led to delays in treatment initiation or failure to treat some patients entirely, the consequences would be particularly dire for patients with the lowest CD4 counts, who have the highest risk of mortality and secondary transmission between HIV testing and treatment initiation 17, 18, 19. Modelling has shown that extended treatment delay among these patients could lead to a decline in population health even as overall numbers on treatment increase 20, 21.

Several studies have found that earlier ART initiation for patients with CD4 between 200 and 350 decreases rates of death, disease progression and incident tuberculosis while increasing retention in care and virologic suppression, among newly eligible patients in both trial 5, 22 and non‐trial settings 6, 8, 23, 24, 25, 26. However, to our knowledge, no study has evaluated the causal impact of large ART eligibility expansions on previously eligible patients. One study in Rwanda found an increase in the median CD4 count at enrolment in care following extension of ART eligibility to patients with CD4 ≤ 350 cells/μl, but did not examine the specific impact on the sickest patients 27. Another study in rural KwaZulu‐Natal, South Africa, found that treatment initiation within 3 months of first clinic visit did not change for the sickest patients after this guideline change 28; however, this analysis did not control for long‐term secular trends. While crowd‐out effects seem plausible under a test‐and‐treat approach, there is no evidence to support this notion, even from previous guideline changes.

To evaluate the potential spillover effects of eligibility expansions, we assessed whether South Africa's expansion from CD4 ≤ 200 to CD4 ≤ 350 cells/μl led to changes in care‐seeking and ART uptake in three groups of patients: those who became eligible under the guideline change (CD4 201–350), the sickest who were eligible both before and after the guideline change (CD4 ≤ 200) and those who were not eligible by CD4 count either before or after the guideline change (CD4 > 350).

## Methods

### Study design and data

We conducted an observational cohort study using data from the Hlabisa HIV Treatment and Care Programme located in KwaZulu‐Natal, South Africa. The Hlabisa programme was initiated in 2004 as part of a public sector roll‐out of HIV treatment in South Africa, implemented as a joint initiative by the Department of Health and the Africa Health Research Institute (formerly the Africa Centre for Population Health). This programme includes 17 primary healthcare clinics throughout the Hlabisa subdistrict that provide HIV rapid testing, counselling and free ART for anyone eligible under national treatment guidelines 29. The programme serves an estimated 228 000 people 30 in a poor, predominantly rural area where adult HIV prevalence was about 29% in 2011 31. The cohort covers the entire subdistrict health system and is the primary source of HIV care and treatment for residents of this area.

Any individual who attends a Hlabisa clinic and tests HIV‐positive has blood drawn for CD4 testing. That same day, the sample is transported to the district hospital laboratory for analysis, and the patient schedules a follow‐up visit at the local clinic for the following week to learn his or her CD4 results. ART eligibility, assessed according to the South African national guidelines, has changed over time: CD4 ≤ 200 cells/μl or WHO stage IV from 2004 32; expansion to people with CD4 ≤ 350 cells/μl if tuberculosis‐infected or pregnant in April 2010 33; all adults with CD4 ≤ 350 cells/μl in August 2011 34; all adults with CD4 ≤ 500 cells/μl in January 2015 35; and universal treatment regardless of CD4 count in September 2016 9. Before initiating ART, patients attend three ART literacy sessions within a 2‐week period unless their health status qualifies them for fast‐tracked treatment initiation. Individuals not yet eligible for ART are encouraged to return to the clinic every 6 months for monitoring.

Through linkage with the National Health Laboratory Service, the Hlabisa programme keeps electronic records for all patients from the time of first CD4 count (facility‐based HIV diagnosis) rather than from the time of ART initiation. Data on sex, age, visit dates, CD4 test results and date of transfer to another clinic (where applicable, if known) are recorded for all patients. Additional data are available for patients who initiate ART, including clinical information, pregnancy and tuberculosis status, and routine laboratory results.

This secondary analysis of deidentified data was determined to be ‘not human subjects research’ by the Boston University Medical Campus Institutional Review Board (protocol H‐35385).

### Outcomes and exposures

We evaluated four outcomes to assess changes in clinic burden and treatment initiation in the period before compared to the period after the August 2011 ART eligibility expansion: (i) monthly number of new HIV diagnoses; (ii) monthly number of patients initiating ART; (iii) monthly proportion of new patients who initiate ART within 6 months; and (iv) days from first clinic visit to treatment initiation. Our analyses include all adults ≥16 years old who sought care in the public sector between 1 April 2010, when the previous guideline change was implemented, and 31 June 2012, 6 months prior to completion of data collection. Outcomes 1 and 2, the numbers of patients entering care and initiating ART, arise from an unobserved reference population of persons in need of HIV care residing in the Hlabisa catchment area. Any smooth secular trends identified by our time series models for these two outcomes likely reflect changes in this implicit denominator. Outcomes 3 and 4 were defined only for HIV‐diagnosed patients who had a CD4 blood sample drawn. Patients were included in these analyses regardless of ART eligibility. We excluded a small number of patients whose ART initiation date preceded their first reported visit to the Hlabisa clinics or who were missing results for their first CD4 test.

All analyses were stratified by CD4 count at the time of facility‐based HIV diagnosis. We defined patients with CD4 ≤ 200 cells/μl as ‘always eligible’ (as they would have been eligible for ART initiation under the 2010 guidelines), patients with CD4 201–350 cells/μl as ‘newly CD4‐eligible’ (as they would not have been eligible until 2011 without another condition) and those with CD4 > 350 cells/μl as ‘never CD4‐eligible’ (as they would not have been eligible before or after the 2011 expansion without another condition). Because CD4 counts were not the sole determinant of ART eligibility, we would expect a slightly diminished effect of the August 2011 expansion among the ‘newly CD4‐eligible’ if any patients were previously eligible due to other conditions. This other eligibility criterion would not have affected the ‘always eligible’ population.

### Statistical analysis

To describe trends in new patient volume and new ART initiator volume, we report the mean monthly count of new HIV diagnoses and new initiators before *vs*. after the policy expansion. To estimate the impact of the reform on (i) numbers seeking care, (ii) numbers starting ART and (iii) the proportion of patients who initiated ART within 6 months, we performed linear regression on monthly values of these three outcomes as interrupted time series analyses controlling for first‐order temporal autocorrelation, using patient data from April 2010 through June 2012. For outcome 3, we excluded individuals whose first 6 months in care overlapped with the eligibility expansion (i.e. first presented in March through August 2011) to avoid capturing outcomes affected by the expansion for patients diagnosed in the pre‐expansion period. Thus, our analysis of outcome 3 compared patients diagnosed during a pre‐period April 2010 through February 2011 with patients diagnosed during a post‐period September 2011 through June 2012. All regression models include the following covariates: a dichotomous indicator taking the value one if enrolment in care occurred after August 2011, a continuous ‘study month’ variable to capture secular trends and a dichotomous ‘holiday’ term controlling for a known pattern of annual changes in clinic attendance in December and January. In alternate models, we added an interaction term between ‘study month’ and the post‐expansion indicator, allowing for separate time trends on either side of August 2011. Under this approach, the post‐expansion indicator estimates the impact immediately following the guideline change, rather than the average effect across the whole post‐expansion period.

To evaluate the impact of the guideline change on outcome 4, days from presentation to treatment initiation, we estimated Cox proportional hazards regression models, controlling for all covariates listed above as well as sex and age. Person‐time was censored at transfer to another clinic outside the Hlabisa system or 180 days of follow‐up. We report adjusted hazard ratios for the effect of presenting for care after the guideline change. Additionally, to assess transitions to ART initiation nonparametrically, we estimated Kaplan–Meier failure curves, comparing patients presenting for care in the pre‐ and post‐periods, adjusting for ‘holiday’, age and sex using inverse probability of treatment weighting methods. All analyses were stratified by CD4 count at clinical presentation: 0–200, 201–350 and >350 cells/μl.

## Results

### Study population

Our study sample included 13 809 patients (after excluding 166 whose ART initiation date preceded their first reported visit to the Hlabisa clinics and 16 without values for first CD4 count). A total of 8531 patients were included prior to the guidelines change as they had their first visit in the 16‐month period 1 April 2010 to 31 August 2011, while 5278 patients were included after eligibility expansion as they first presented in the 10‐month period 1 September 2011 to 30 June 2012. Patients were predominantly female (68%), with a median age of 31 years at first presentation (IQR 25–39; Table 1).

**Table 1:** Characteristics of patients seeking HIV care in Hlabisa, KwaZulu‐Natal, South Africa

	April 2010–August 2011 (*n* = 8531)	September 2011–June 2012 (*n* = 5278)	Total (*n* = 13 809)
First CD4 count, *n* (%)			
CD4 < 200	3455 (40.5%)	1595 (30.2%)	5050 (36.6%)
CD4 200–350	2188 (25.6%)	1422 (26.9%)	3610 (26.1%)
CD4 > 350	2888 (33.9%)	2261 (42.8%)	5149 (37.3%)
Started ART within 6 months, *n* (%)			
Overall	2659 (31.2%)	1815 (34.4%)	4474 (32.4%)
CD4 < 200^a^	2005 (58.0%)	1008 (63.2%)	3013 (59.7%)
CD4 200–350^a^	570 (26.1%)	710 (49.9%)	1280 (35.5%)
CD4 > 350^a^	84 (2.9%)	97 (4.3%)	181 (3.5%)
Female, *n* (%)	5784 (67.8%)	3589 (68.0%)	9373 (67.9%)
History of TB at ART initiation, *n* (%)^b^	659 (24.8%)	314 (17.3%)	973 (21.7%)
Age in years at first CD4 count, median (IQR)	31 (25–39)	31 (25–39)	31 (25–39)
Age in years at ART initiation, median (IQR)	32 (27–40)	32 (26–40)	32 (26–40)

ART, antiretroviral therapy; TB, tuberculosis; IQR, interquartile range.

a Denominator for % is number of individuals from this category of ‘First CD4 count’.

b Denominator for % is number of individuals from this category who initiated ART within 6 months.

At first CD4 count, 5050 patients (37%) fell into the ‘always eligible’ CD4 category (≤200 cells/μl), 3610 (26%) were ‘newly CD4‐eligible’ (201–350 cells/μl), and 5149 (37%) were ‘never CD4‐eligible’ (>350 cells/μl). Overall, 4474 patients (32%) initiated ART within 6 months from first presentation, including patients not yet eligible for ART.

### Number of patients seeking HIV care

**Figure 1:**
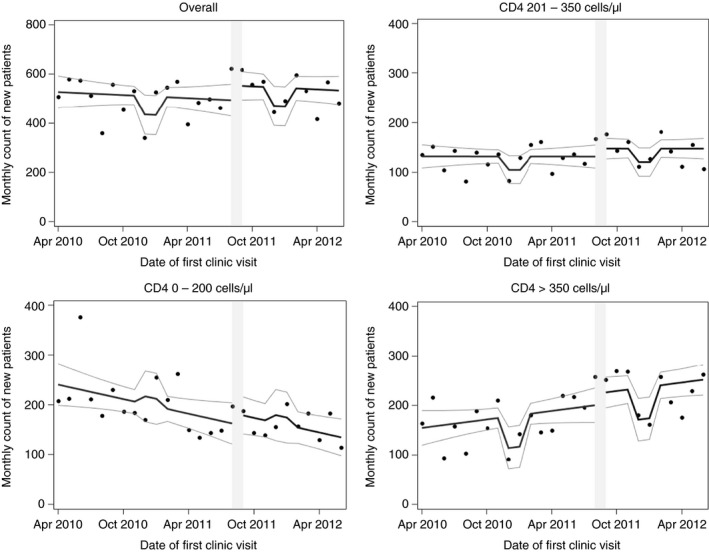
Observed monthly count of new diagnoses over time at the Hlabisa HIV Treatment and Care Programme clinics in KwaZulu‐Natal, South Africa, along with curve of predicted values and 95% confidence limits. Predicted values based on the following model: *Count = β*_*0*_ *+ β*_*1*_ *(study month) + β*_*2*_ *(guideline change) + β*_*3*_ *(holiday)* Figure is stratified by CD4 count at clinical presentation. Grey bar indicates month of policy implementation.

While eligibility expansion increases the proportion of patients eligible for treatment, it might also impact the number of patients presenting for HIV testing if they believe they would be more likely to access treatment if positive. An average of 501.8 (SD = 77.8) patients diagnosed per month before the guideline change increased to 527.8 (SD = 65.6) after the guideline change, a crude change of +26.0 (95% CI: −34.5; 86.4; Table 2). However, controlling for the long‐term decline in new diagnoses (Figure 1) revealed that the policy increased patient presentation by 62.7 per month (95% CI: −24.6; 150.0) or 12.5%, relative to expectation (Table 2). Our adjusted analyses stratified by first CD4 count showed an immediate increase in the numbers of ‘newly CD4‐eligible’ and ‘never CD4‐eligible’ patients seeking care following the eligibility expansion [+34.1; 95% CI (1.1; 67.1) and +58.8; 95% CI (3.3; 114.4), respectively] (Appendix 1). However, this did not lead to long‐term changes in average patient volume (Figure 1, Table 2).

**Table 2:** Effect of August 2011 eligibility expansion on entry into care and ART uptake among patients previously eligible, newly eligible and not yet eligible

CD4 count at clinical diagnosis	Number entering care per month				Number initiating ART per month				Proportion initiating ART at 6 months^a^				Time to ART initiation	
	Pre, mean (SD)	Post, mean (SD)	Crude ∆, 95% CI	Adjusted ∆, 95% CI^b^	Pre, mean (SD)	Post, mean (SD)	Crude ∆, 95% CI	Adjusted ∆, 95% CI^b^	Pre, % (SE)^a^	Post, % (SE)	Crude ∆, 95% CI	Adjusted ∆, 95% CI^b^	Crude HR, 95% CI	Adjusted HR, 95% CI^c^
Overall	501.8 (77.8)	527.8 (65.6)	26.0 (−34.5; 86.4)	62.7 (−24.6; 150.0)	300.5 (61.9)	378.5 (74.2)	78.0 (23.4; 132.7)	95.5 (−1.3; 192.3)	32.4 (0.6)	34.4 (0.7)	1.9 (0.2; 3.7)	−0.9 (−12.4; 10.7)	1.18 (1.11; 1.26)	1.18 (1.06; 1.33)
Previously eligible: CD4 ≤ 200	203.2 (57.8)	159.5 (28.6)	−43.7 (−84.2; −3.2)	22.5 (−44.6; 89.5)	146.1 (30.8)	129.3 (28.0)	−16.8 (−41.2; 7.7)	10.6 (−28.6; 49.8)	57.9 (1.0)	63.2 (−1.2)	5.3 (2.2; 8.4)	4.0 (−2.7; 10.6)	1.29 (1.20; 1.40)	1.06 (0.92; 1.23)
Newly eligible: CD4 201–350	128.7 (25.9)	142.2 (27.2)	13.5 (−8.2; 35.1)	15.5 (−18.4; 49.4)	85.8 (19.1)	142.6 (26.8)	56.8 (38.6; 75.0)	73.0 (42.1; 103.9)	24.3 (1.2)	49.9 (1.3)	25.6 (22.2; 29.1)	5.5 (−8.8; 19.8)	2.42 (2.16; 2.71)	1.47 (1.19; 1.82)
Not yet eligible: CD4 > 350	169.9 (47.3)	226.1 (41.7)	56.2 (19.0; 93.5)	22.3 (−41.1; 85.7)	56.2 (18.9)	92.9 (24.9)	36.7 (19.2; 54.2)	30.3 (−6.9; 67.6)	2.9 (0.4)	4.3 (0.4)	1.4 (0.3; 2.6)	−0.7 (−2.4; 1.0)	1.49 (1.11; 2.00)	1.19 (0.69; 2.06)

ART, antiretroviral therapy; CI, confidence interval; SD, standard deviation; SE, standard error; HR, hazard ratio; ∆, Change from ‘Pre’ to ‘Post’.

a Excluding patients diagnosed from March 2010 through August 2011 whose first 6 months as patients overlap with eligibility expansion.

b Linear regression controlling for first‐degree autocorrelation, adjusted for continuous ‘study month’ and dichotomous ‘holiday’ term.

c Cox proportional hazards regression adjusted for continuous ‘study month’, dichotomous ‘holiday’ term, sex and age.

### Number of patients initiating ART

The average number of patients initiating ART per month increased from 300.5 (SD = 61.9) before the guideline change to 378.5 (SD = 74.2) afterwards (Table 2). Our adjusted linear regression revealed that, because initiation had been declining over time, the average monthly increase in new ART initiators attributable to the policy change was 95.5 (95% CI: −1.3; 192.3), a 32% increase over the adjusted pre‐period average (Table 2).

**Figure 2:**
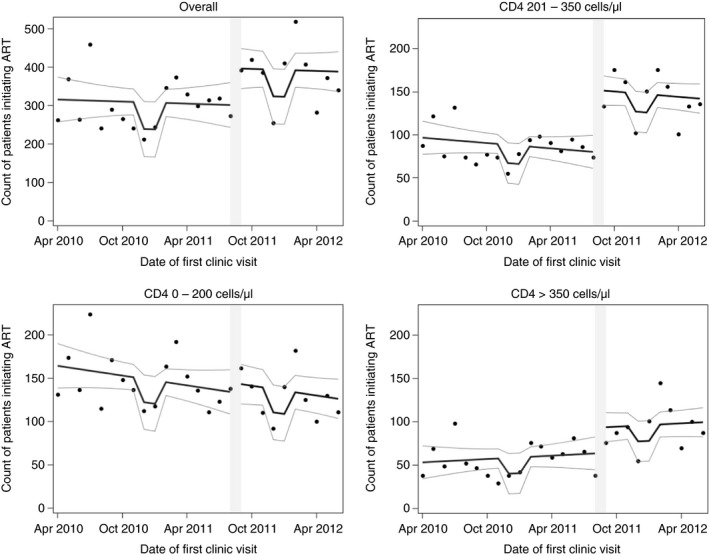
Observed monthly count of new ART initiators over time at the Hlabisa HIV Treatment and Care Programme clinics in KwaZulu‐Natal, South Africa, along with curve of predicted values and 95% confidence limits. Predicted values based on the following model: *Count = β*_*0*_ *+ β*_*1*_ *(study month) + β*_*2*_ *(guideline change) + β*_*3*_ *(holiday)* Figure is stratified by CD4 count at clinical presentation. Grey bar indicates month of policy implementation. ART, antiretroviral therapy.

When stratified by first CD4 count, this increase in ART initiation was most pronounced among ‘newly CD4‐eligible’ patients (CD4 201–350), who averaged 85.8 (SD = 19.1) initiators per month prior to the guideline change and 142.6 (SD = 142.6) afterwards, an adjusted increase of 73.0 patients per month [95% CI (42.1; 103.9)] or 85% (Table 2). We found evidence of a small increase among patients with CD4 > 350 (‘never CD4‐eligible’) [adjusted +30.3; 95% CI (−6.9; 67.6)]. There was no evidence of a decline in the number of monthly ART initiators among sicker patients who were always eligible (CD4 ≤ 200 cells/μl). Figure 2 plots trends in number of ART initiators by CD4 count.

### Time from clinical HIV diagnosis to ART initiation

Because these data were collected in the era prior to universal treatment, monthly numbers of ART initiators are the product of two factors: numbers entering care (presented above) and rates of progression to ART initiation. The proportion of patients who started ART within 6 months of HIV diagnosis doubled in the ‘newly CD4‐eligible’ group from 24.3% to 49.9% [+25.6 (95% CI: 22.2; 29.1)] when comparing April 2010 through February 2011 *vs*. the 10 months following the policy change. Among ‘always eligible’ patients (CD4 ≤ 200), there was no evidence of impeded transition to ART, with the proportion initiating within 6 months increasing slightly from 57.9% pre‐reform to 63.2% post‐reform [crude +5.3 percentage points (95% CI: 2.2; 8.4)]. Similarly, the proportion of ‘never CD4‐eligible’ patients (CD4 > 350) who initiated within 6 months increased modestly from 2.9% to 4.3% [crude +1.4 percentage points (95% CI: 0.3; 2.6)] (Table 2). These findings were confirmed, albeit attenuated, after controlling for time trends, with adjusted increases of 4.0 percentage points (95% CI: −2.7; 10.6) for the ‘always eligible’, 5.5 percentage points (95% CI: −8.8; 19.8) for the ‘newly CD4‐eligible’ and −0.7 percentage points (95% CI: −2.4; 1.3) for the ‘never CD4‐eligible’ categories (Table 2). Models of immediate impact, with different slopes after the policy change, revealed similar results (Appendix 1). Appendix 2 plots changes in 6‐month initiation by CD4 count.

Moving from a binary treatment indicator to a continuous‐time survival analysis, we estimated adjusted Cox proportional hazards models. The overall speed of ART initiation increased following the guideline change (HR = 1.18; 95% CI 1.06; 1.33). This increase was strongest among ‘newly CD4‐eligible’ patients [HR = 1.47; 95% CI (1.19; 1.82)], with possible modest increases among the ‘always eligible’ (HR = 1.06; 95% CI 0.92; 1.23) and ‘never eligible’ categories (HR = 1.19; 95% CI 0.69; 2.06; Table 2). We found similar results when focusing on the period immediately following the policy change (Appendix 1).

**Figure 3:**
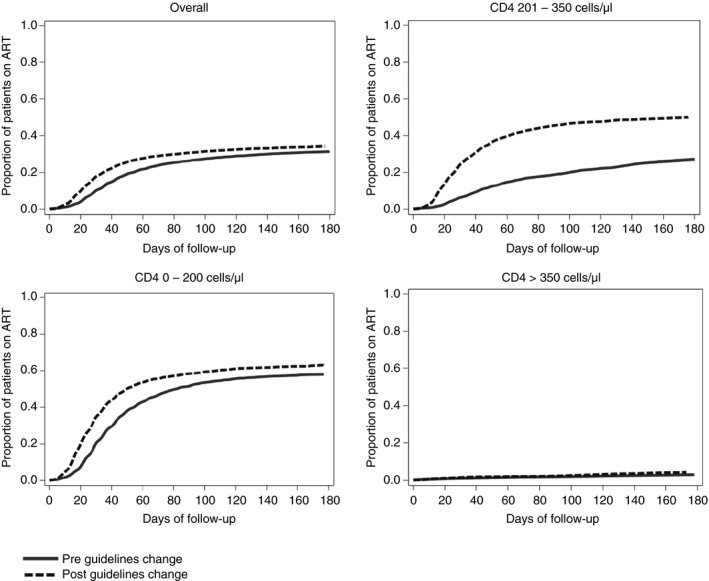
Kaplan–Meier curves showing the cumulative probability of treatment initiation before (solid) and after (dashed) the guideline change, adjusted for holiday, sex and age using inverse probability of treatment weighting. Figure is stratified by CD4 count at clinical presentation. ART, antiretroviral therapy.

To illustrate the progression of patients from diagnosis to treatment before and after the eligibility expansion, Figure 3 shows Kaplan–Meier failure curves adjusted for holiday, sex and age (but, notably, not for the underlying time trend). The figures reveal four important trends. First, there was a very large increase in the proportion initiating ART among ‘newly CD4‐eligible’ patients. Second, among ‘always eligible’ patients (CD4 ≤ 200 cells/μl), there was no evidence of inferior outcomes post‐expansion; in fact, the proportion initiating increased somewhat for this group. Third, among ‘always eligible’ patients who initiated ART, time from diagnosis to initiation fell from a median of 40 days (IQR: 27; 63) to 28.5 days (IQR: 18; 44). Although the sickest patients initiated faster, patients in the newly CD4‐eligible category progressed to initiation within a time frame similar to the sickest pre‐reform. Finally, although the reform increased initiation, the proportion initiating ART among eligible patients remained relatively low.

## Discussion

Expanding CD4 eligibility criteria for ART to ≤350 cells/μl at the Hlabisa HIV Treatment and Care Programme in rural South Africa resulted in an 85% increase in ART initiation among ‘newly eligible’ patients with CD4 counts between 200 and 350 cells/μl and a 32% increase in the number of patients initiating ART overall. In spite of this large influx of healthier patients onto ART, we found no evidence of crowd‐out among sicker patients who have the greatest need for ART. In fact, we found that ART uptake increased modestly for the sickest patients and times to initiation fell. Maintaining the timeliness of treatment is critical, especially for the sickest patients, who show the greatest rise in mortality when not treated promptly 17, 18, 19.

In addition to faster progression from diagnosis to ART initiation, we found some evidence that raising CD4 thresholds led to increased care‐seeking, at least in the short run, a phenomenon that has been reported elsewhere 27. We found the greatest increase in new patients at higher CD4 counts, who would not likely have been eligible before the policy change. These results suggest that extending treatment eligibility to all HIV‐infected individuals could increase the proportion of HIV‐infected individuals who seek HIV testing, which would in turn increase numbers on treatment. The gains in numbers seeking care appeared to be short‐lived, however, perhaps reflecting publicity around the guideline change or depletion of the pool of untreated eligible individuals after a shift of new patients into care. Interestingly, we found no increase in new patients in the previously eligible (CD4 ≤ 200 cells/μl) category, suggesting that beliefs about the probability of being eligible for treatment may be an important factor in HIV testing and care‐seeking behaviour.

Increasing the number of patients on treatment without compromising timeliness of care is a noteworthy achievement and may bode well for the rising patient loads as South Africa implements ‘test‐and‐treat’. Indeed, the eligibility expansion was implemented successfully in Hlabisa, despite being located in one of the poorest districts in South Africa 36, in a predominantly rural area with very high HIV prevalence 31. Several factors may explain our findings. First, there may have been more efficient implementation of nurse‐initiated management of ART during this period 37. Second, clinics fast‐tracked the sickest patients for accelerated initiation and initiated healthier patients on a less urgent schedule to maximise the potential benefit of existing resources. These differences can be observed in the reduction in time to initiation for patients with CD4 ≤ 200 cells/μl pre‐ *vs*. post‐August 2011, as well as the shorter time to initiation for patients with CD4 ≤ 200 cells/μl relative to healthier patients with CD4 > 200 cells/μl post‐August 2011 (Figure 3). Further research is needed to determine the relative contributions of these factors to the capacity to take on more patients and to determine more precisely the lessons for health systems in other settings.

Our results should be considered in the light of their limitations. We evaluated the experience of one system of clinics in one region of South Africa, and our results may not generalise to other settings that experience different drivers into ART initiation or different systemic barriers to expanding care. The statistical power of our analysis was also limited, as our data on ART uptake were complete only through December 2012. This constrained the post‐eligibility expansion period to 10 months after the guideline change (through June 30, 2012) for our outcome of interest, ART initiation within 6 months. Additionally, time series analyses are vulnerable to confounding by unpredictable (and unmodelled) secular trends. However, our exclusion of data that would be affected by earlier policy changes, our adjustment for changes in clinic operations during the holiday season and our control for gradual changes over time mitigate against such biases. Finally, we assessed the spillover effect of the eligibility expansion on entry into care and time to ART initiation among patients with lower (previously eligible) CD4 counts. However, additional dimensions of quality that were not observed in this study would be an important avenue for future research. For example, it has been documented that timely switching of ART to second‐line therapy based on viral load failure is suboptimal, possibly because of time pressure on clinic staff 38.

Our finding that the Hlabisa HIV Treatment and Care Programme successfully implemented ART eligibility scale‐up without incurring negative spillover effects on the sickest patients provides support for the feasibility of further programme expansion. This particular programme can serve as a model for scale‐up in other rural, resource‐limited settings and should be considered when evaluating the economic costs and implications of ART eligibility expansions. As South Africa has now extended treatment to all HIV‐infected patients, it will be important to continue to monitor the impact of these policies on all patients across CD4 levels and throughout the treatment cascade.

## Appendix 1 Immediate effects of eligibility expansion on entry into care and ART uptake

**Table:**

CD4 count at clinical diagnosis	Intercept shift immediately following the guideline change^a^			
	Number entering care per month	Number initiating ART per month	Proportion initiating ART at 6 months	Time to ART initiation (HR)
	Adjusted change (95% CI)^b^	Adjusted change (95% CI)^b^	Adjusted change (95% CI)^b^	Adjusted change (95% CI)^b^
Overall	97.2 (−0.2; 194.6)	117.6 (7.2; 228.0)	1.6 (−7.8; 10.9)	1.08 (0.95; 1.22)
Previously eligible: CD4 ≤ 200	10.2 (−66.2; 86.6)	16.0 (−30.1; 62.0)	4.6 (−2.3; 11.5)	1.08 (0.92; 1.27)
Newly eligible: CD4 201–350	34.1 (1.1; 67.1)	81.2 (46.6; 115.8)	10.8 (1.0; 20.6)	1.38 (1.11; 1.73)
Not yet eligible: CD4 > 350	58.8 (3.3; 114.4)	28.9 (−14.1; 71.9)	−0.4 (−2.2; 1.3)	1.12 (0.63; 2.0)

Notes

ART, antiretroviral therapy; CI, confidence interval; HR, hazard ratio.

a Linear regression controlling for first‐degree autocorrelation, with the following independent variables: a dichotomous policy change indicator (coefficient shown), continuous ‘study month’, dichotomous ‘holiday’ indicator, and interaction of ‘study month’ with the policy change indicator. Effect estimate is the change in intercept at the time of the policy change, allowing for separate slopes before and after.

b Confidence intervals estimated using bootstrapping with 1000 replicates.

## Appendix 2

Observed monthly per cent of new patients who initiate ART within 6 months of first visit at the Hlabisa HIV Treatment and Care Programme clinics in KwaZulu‐Natal, South Africa, along with curve of predicted values. Predicted values based on the following model: *% who initiate by 6 months = β*_*0*_ *+ β*_*1*_ *(study month) + β*_*2*_ *(guideline change) + β*_*3*_ *(holiday)* (Model for figure does not account for autocorrelation). Grey segment indicates months excluded from regression model due to patients’ exposure to both sets of guidelines in first 6 months in HIV care. ART, antiretroviral therapy.
